# Review of phytomedicine, phytochemistry, ethnopharmacology, toxicology, and pharmacological activities of Cymbopogon genus

**DOI:** 10.3389/fphar.2022.997918

**Published:** 2022-08-29

**Authors:** Jonnea Japhet Tibenda, Qiong Yi, Xiaobo Wang, Qipeng Zhao

**Affiliations:** ^1^ School of Pharmacy, Key Laboratory of Hui Ethnic Medicine Modernization, Ministry of Education, Ningxia Medical University, Yinchuan, China; ^2^ Meishan Traditional Chinese Medicine Hospital, Meishan, China; ^3^ Research Institute of Integrated TCM and Western Medicine, Chengdu University of Traditional Chinese Medicine, Chengdu, China

**Keywords:** Cymbopogon, phytomedicine, phytochemistry, essential oils, pharmacological activity

## Abstract

The Cymbopogon genus belongs to the Andropoganeae family of the family Poaceae, which is famous for its high essential oil concentration. Cymbopogon possesses a diverse set of characteristics that supports its applications in cosmetic, pharmaceuticals and phytotherapy. The purpose of this review is to summarize and connect the evidence supporting the use of phytotherapy, phytomedicine, phytochemistry, ethnopharmacology, toxicology, pharmacological activities, and quality control of the Cymbopogon species and their extracts. To ensure the successful completion of this review, data and studies relating to this review were strategically searched and obtained from scientific databases like PubMed, Google Scholar, ResearchGate, ScienceDirect, and Elsevier. Approximately 120 acceptable reviews, original research articles, and other observational studies were included and incorporated for further analysis. Studies showed that the genus Cymbopogon mainly contained flavonoids and phenolic compounds, which were the pivotal pharmacological active ingredients. When combined with the complex β-cyclodextrin, phytochemicals such as citronellal have been shown to have their own mechanism of action in inhibiting the descending pain pathway. Another mechanism of action described in this review is that of geraniol and citral phytochemicals, which have rose and lemon-like scents and can be exploited in soaps, detergents, mouthwash, cosmetics, and other products. Many other pharmacological effects, such as anti-protozoal, anti-bacterial, anti-inflammatory and anti-cancer have been discussed sequentially, along with how and which phytochemicals are responsible for the observed effect. Cymbopogon species have proven to be extremely valuable, with many applications. Its phytotherapy is proven to be due to its rich phytochemicals, obtained from different parts of the plant like leaves, roots, aerial parts, rhizomes, and even its essential oils. For herbs of Cymbopogon genus as a characteristic plant therapy, significant research is required to ensure their efficacy and safety for a variety of ailments.

## Graphical Abstract

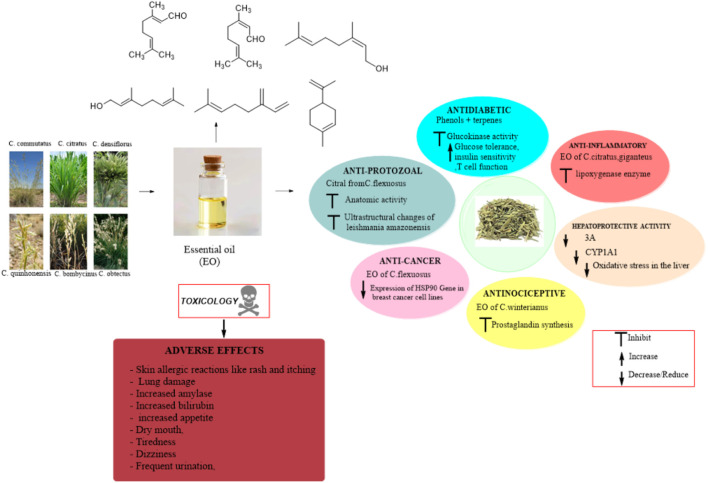

## Introduction

**FIGURE 1:**
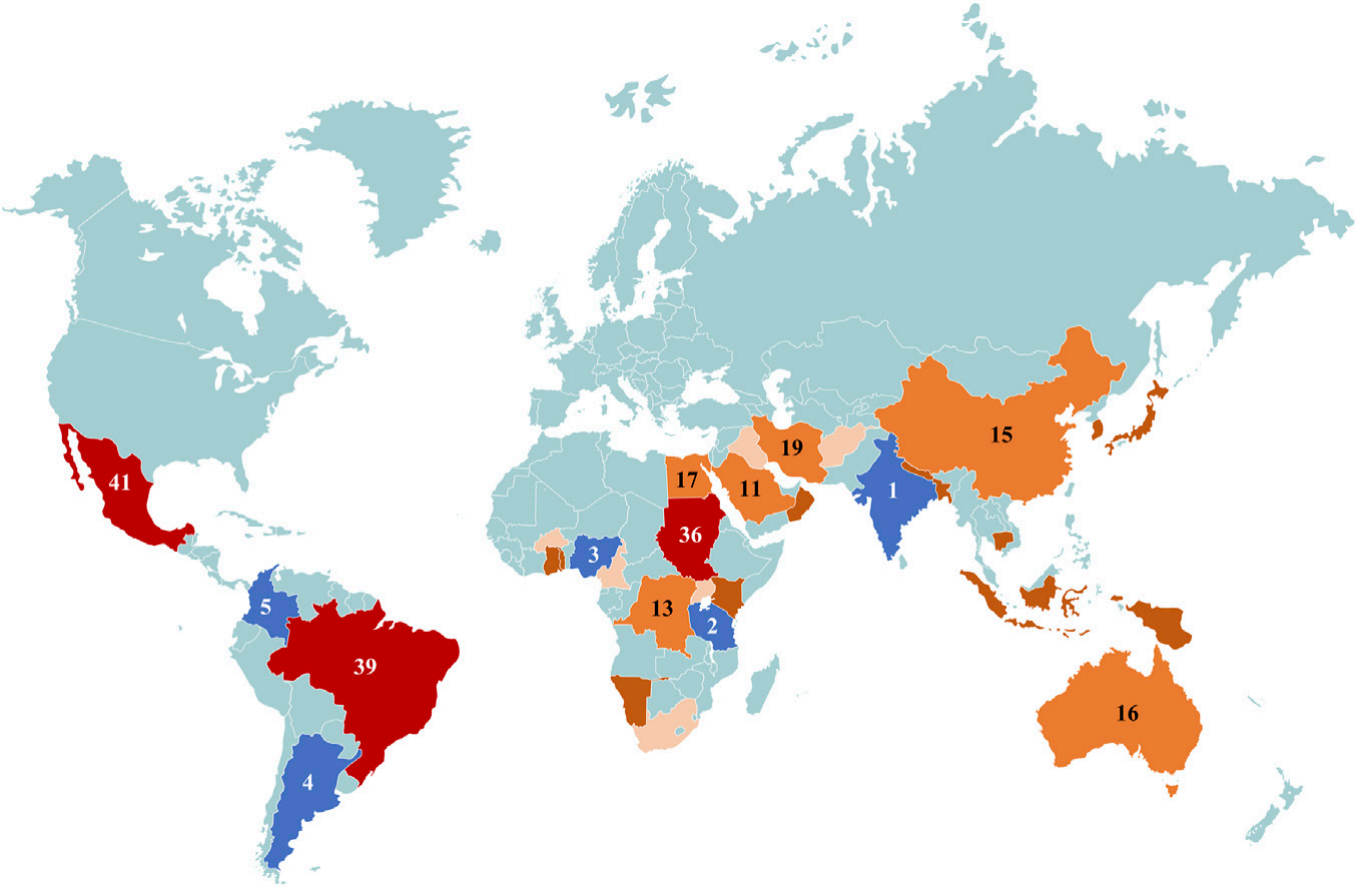
The distribution of Cymbopogon species from different countries across the world, with more than 10 species found in countries like India, China, Australia and countries found in East Africa.

**FIGURE 2:**
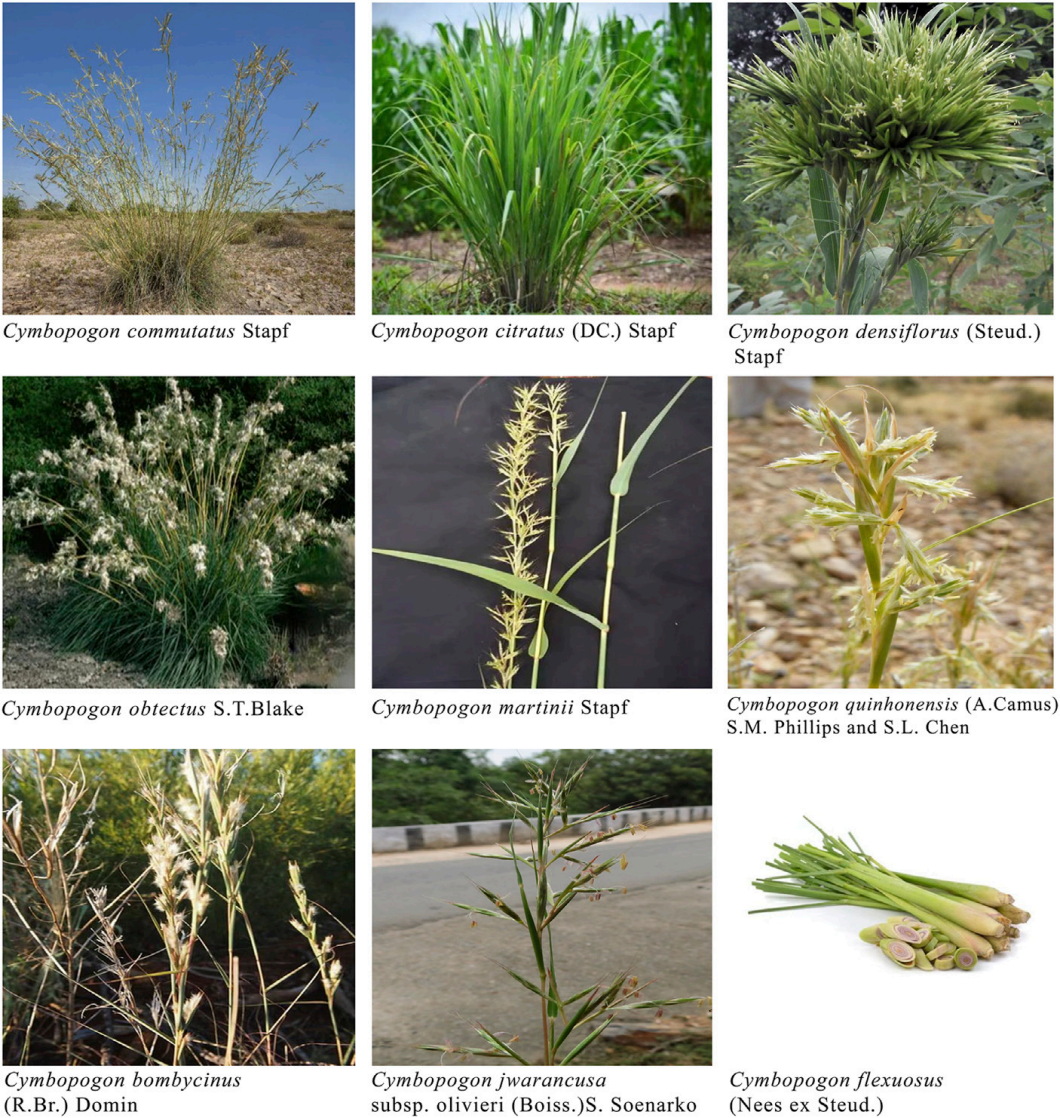
Depiction of diversiform Cymbopogon species.

Cymbopogon is a genus with a lot of names, such as lemongrass, barbed wire grass, silky heads, Cochin grass, Malabar grass, oily heads, citronella grass, or fever grass. This grass species is widely distributed in more than 40 countries in the world (as shown in Figure 1) and is native to Asia, Africa, Australia, and tropical islands (Soenarko 1977; Toungos 2019). Cymbopogon is diverse in terms of names, species, and uses, with almost all of them being aromatic. It consists of 144 species, some of which include *Cymbopogon nardus* (L.) Rendle (C. nardus), *Cymbopogon citratus* (DC.) Stapf (C. citratus), *Cymbopogon giganteus* Chiov (C. giganteus), *Cymbopogon flexuosus* (Nees ex Steud.) W. Watson (C. flexuous), *Cymbopogon martini* (Roxb.) W. Watson (C. martinii), *Cymbopogon schoenanthus* subsp. proximus (Hochst. ex A. Rich.) Maire & Weiller (C. schoenanthus), etc. (Table 1 and Figure 2). The distribution of these species is astonishing as some of the same species can be found in different countries, but there no conclusive evidence to indicate whether or not their chemical composition is exactly the same. For example, C. citratus can be found in Bangladesh, Brazil, Tanzania, Ghana, Guadeloupe, French West Indies, Kenya, Mauritius, Argentina, Thailand, Uganda, Nigeria, India, Mexico, Singapore, Togo, Trinidad, etc. C. nardus can be hunted in Uganda, Brazil, Mauritius, Thailand, etc. While C. giganteus can be found in Ghana, Nigeria, Cameroon, Burkina Faso, Madagascar, etc. And C. jwarancusa can be found in Iran, Pakistan, etc (Jirovetz et al., 2007; Karami et al., 2021). The distribution of Cymbopogon species from different countries across the world is shown in Figure 1.

**TABLE 1:** Cymbopogon species and sections of the plant or essential oil are used for traditional, medicinal, and economic use.

Species	Known names	Place	Plant section	Reference
*Cymbopogon nardus* (L.) Rendle	Ceylon citronella	India	Leaves	Noor et al. (2012)
*Cymbopogon citratus* (DC.) Stapf	Lemon grass and Limonaria grass tea	Tanzania, Nigeria, India, Argentina, Columbia and Costa Rica	Leaves, aerial and rhizomes	Santos et al. (2013)
				Moreira et al. (2010)
*Cymbopogon giganteus* Chiov.	Tsauri grass and ahibero	Cameroon, Burkina Faso and Madagascar	Leaves and flowers	Jirovetz et al. (2007)
*Cymbopogon flexuosus* (Nees ex Steud.) W. Watson	Lemongrass	India	Leaves and rhizomes	Desai and Parikh (2012)
*Cymbopogon martini* (Roxb.) W. Watson	Palmarosa grass	Indian, Myanmar and Vietnam	Leaves	KWCSPF
*Cymbopogon schoenanthus* (L.) Spreng.	Ethkher and camel grass	Saudi Arabia	Leaves	AI-Ghamdi et al. (2007)
*Cymbopogon densiflorus* (Steud.) Stapf	Lemongrass	Congo	Leaves	De Smet (1996): Ekpenyong et al. (2015)
*Cymbopogon excavatus* (Hochst.) Stapf ex Burtt Davy	Bread-leavened and turpentine grass	South Africa	Flowers	Govere et al. (2000)
*Cymbopogon parkeri* Stapf	Lemon grass	Pakistan	Leaves	Bagheri et al. (2007)
*Cymbopogon Validus* (Stapf) Stapf ex Burtt Davy	African bluegrass	Eastern and southern Africa	Leaves	Kepe (2004)
*Cymbopogon refractus* (R.Br.) A. Camus	Barbed wire grass	Australia	Essential oils	Avoseh et al. (2015)
*Cymbopogon obtectus* S.T. Blake	Silky-heads	Central Australia	Leaves	
*Cymbopogon liangshanensis* S.M. Phillips & S.L. Chen	Liangshan Xiangmao (Liangshan citronella)	Sichuan	Leaves	Phillips and Hua (2005)
*Cymbopogon tungmaiensis* L. Liu	Tongmai Xiangmao (Lemon grass)	Sichuan, Tibet and Yunnan	Leaves	
*Cymbopogon jwarancusa* (Jones ex Roxb.) Schult.	Lemon grass	Egypt	Aerials	El-Bakry and Abdel-Salam (2012)
*Cymbopogon commutatus* (Steud.) Stapf	Incense grass, aromatic rush, camel’s hay and lemon grass	Sahel, East Africa, Arabian Peninsula, Iraq, Iran, Afghanistan, India and Pakistan	Essential oils	Mandaville (2013); KWCSPF
*Cymbopogon ambiguus* (Hack.) A. Camus	Australian lemon-scented grass	Australia and Timor	Essential oils	KWCSPF
*C. annamensis* A. Camus	A. Camus	Yunnan, Laos, Vietnam and Thailand	Leaves	KWCSPF
*Cymbopogon bhutanicus* Noltie	——	Bhutan	Leaves	KWCSPF
*Cymbopogon bombycinus* (R.Br.) Domin	Silky oil grass	Australia	Leaves	KWCSPF
*Cymbopogon clandestinus* Stapf	Limestone and citronella	Thailand and Kedah	Essential oils	KWCSPF
*Cymbopogon winterianus* Jowitt ex Bor	Citronella grass	Borneo, Java and Sumatra	Essential oils and stem	Andila et al. (2018)
*Cymbopogon xichangensis* R. Zhang & B.S. Sun	——	Sichuan	Leaves	KWCSPF
*Cymbopogon dieterlenii* Stapf ex Schweick.	——	Lesotho, Namibia and South Africa	——	KWCSPF
*Cymbopogon marginatus* (Steud.) Stapf ex Burtt Davy	Dobograss	Cape province of South Africa	Leaves	KWCSPF
*Cymbopogon microstachys* (Hook.f.) Soenarko	——	Indian Subcontinent, Myanmar, Thailand and Yunnan	Leaves	KWCSPF
*Cymbopogon microthecus* (Hook.f.) A. Camus	——	Nepal, Bhutan, Assam, West Bengal and Bangladesh	Leaves	KWCSPF
*Cymbopogon minor* B.S. Sun & R. Zhang ex S.M. Phillips & S.L. Chen	——	Yunnan	Leaves	KWCSPF
*Cymbopogon cambogiensis* (Balansa) E.G. Camus & A. Camus	Balansa	Thailand, Cambodia and Vietnam	Leaves	KWCSPF
*Cymbopogon calciphilus* Bor	——	Thailand	Leaves and rhizome	KWCSPF
*Cymbopogon minutiflorus* S. Dransf	——	Sulawesi	Leaf-sheaths	KWCSPF
*Cymbopogon goeringii* (Steud.) A. Camus	——	China, Taiwan, Korea, Japan incl Ryukyu Islands and Vietnam	Essential oils	KWCSPF
*Cymbopogon khasianus* (Hack.) Stapf ex Bor	——	Yunnan, Guangxi, Assam, Bhutan, Bangladesh, Myanmar and Thailand	Essential oils	Choudhury and Leclercq (1995); KWCSPF
*Cymbopogon auritus* B.S. Sun	——	Yunnan	Leaves	TPL
*Cymbopogon caesius* (Hook. & Arn.) Stapf	Kachi grass, common turpentine grass and broad-leaved turpentine grass	Sub-Saharan Africa, Indian Subcontinent, Yemen, Afghanistan, Madagascar, Comoros and Réunion	Mixtures and roots	Leistner (2000)
*Cymbopogon fibrosus* B.S. Sun	Citronella	Sichuan and Yunnan	Leaves	TPL
*Cymbopogon nervosus* B.S. Sun	Delft grass	Yunnan	——	TPL
*C. traninhensis* (A. Camus) Soenarko	Lemon grass and Heng Xiangmao	Yunnan, India, Laos, Myanmar and Thailand	Leaves	
*Cymbopogon proximus* Stapf	Lemon grass	Egypt and Northern parts of Sudan	Essential oil	El Tahir and Abdel-Kader (2008)
*C. queenslandicus* S.T. Blake	Silky head	Australia	——	
*Cymbopogon nervatus* (Hochst.) Chiov.	——	Myanmar, Thailand and central Africa	Leaves	KWCSPF
*Cymbopogon pospischilii* (K. Schum.) C.E. Hubb.	Bitter turpentine grass	Eastern, southern Africa and Oman, Yemen, Himalayas, Tibet and Yunnan	Leaves and aerial	KWCSPF
*Cymbopogon osmastonii* R. Parker	——	India and Bangladesh	Leaves	KWCSPF
*Cymbopogon clandestinus* Stapf	Myetsat (pungent tasting grass)	Thailand, Myanmar and Andaman Islands	Essential oils	KWCSPF
*Cymbopogon coloratus* (Hook.f.) Stapf	——	Madhya Pradesh, Tamil Nadu, Myanmar and Vietnam	Essential oils	
*Cymbopogon pendulus* (Nees ex Steud.) W. Watson	——	Yunnan, eastern Himalayas, Myanmar and Vietnam	Essential oils	
*Cymbopogon polyneuros* Stapf	——	Tamil Nadu, Sri Lanka and Myanmar	Leaves	KWCSPF
Cymbopogon dependens B.K. Simon	——	Australia	Leaves	KWCSPF
*Cymbopogon distans* (Nees ex Steud.) W. Watson	——	Gansu, Guizhou, Shaanxi, Sichuan, Tibet, Yunnan, Nepal, northern Pakistan and Jammu & Kashmir	Essential oils	
*Cymbopogon exsertus* (Hack.) A. Camus	——	Nepal and Assam	Leaves	KWCSPF
*Cymbopogon pruinosus* (Nees ex Steud.) Chiov.	Ahibero	Madagascar	Aerial	Ruphin et al. (2016)
*Cymbopogon procerus* (R.Br.) Domin	Native lemon grass	Australia, New Guinea, Maluku, Lesser Sunda Islands and Sulawesi	Leaves and stems	
*Cymbopogon gidarba* (Steud.) A. Camus	——	Indian Subcontinent, Myanmar and Yunnan	Leaves	KWCSPF
*Cymbopogon rectus* (Steud.) A. Camus	——	Lesser Sunda Islands and Java	Leaves	KWCSPF
*Cymbopogon globosus* Henrard	——	Maluku, New Guinea and Queensland	Leaves	KWCSPF
*Cymbopogon exaltatus* (R.Br.) Domin	Ginger lemon grass	Australia	Essential oils	Akhila (2009)
*Cymbopogon mekongensis* A. Camus	Lemongrass	China	Leaves	

Note:KWCSPF, kew world checklist of selected plant families; TPL, the plant list.

The ethnopharmacology of Cymbopogon species has been shown to possess a diverse set of characteristics that supports their traditionally applications in cosmetic, pharmaceuticals, insect repellants, insecticides, and perfumery, mainly due to their high level of essential oils (Khanuja et al., 2005; Aibinu et al., 2007). Several findings have confirmed the nutritional value of Cymbopogon species as well as their therapeutic and pharmacological properties. Many countries in the world use this species as an herbal remedy on urinary tract infection, detoxication effects on the kidney and liver, bone diseases, hypertension, hypercholesteremia, stomach ulcers, weight loss and indigestion (Takaisi-Kikuni et al., 2000; Jirovetz et al., 2007; Francisco et al., 2011; Kpoviessi et al., 2014; Karami et al., 2021). When it comes to dosage, in countries like Tanzania, it is believed that patients with stomach ulcers can have a cup of tea of this herb half an hour before eating in the morning, afternoon, and evening. In diabetic patients, you boil the lemongrass leaves and add ginger powder and take them before dinner. The doses and how it is consumed vary in other countries, as some consume it as tea or decoction with different dosages. C. citratus is one of the most extensively employed species in the world, with pharmacological effects such as anti-inflammatory, antitrypanosomal, and stomach discomfort treatments (Francisco et al., 2011; Kpoviessi et al., 2014). Other species with antimicrobial effects include C. giganteus (Jirovetz et al., 2007), C. pendulus and C. winterianus as antifungals (Pandey et al., 1996; Oliveira-Verbel et al., 2011), C. flexuosus as a chemo preventive (Sharma et al., 2009), C. densiflorus Stapf as an antibacterial (Takaisi-Kikuni et al., 2000).

## Ethnopharmacology and traditional significance

**FIGURE 3:**
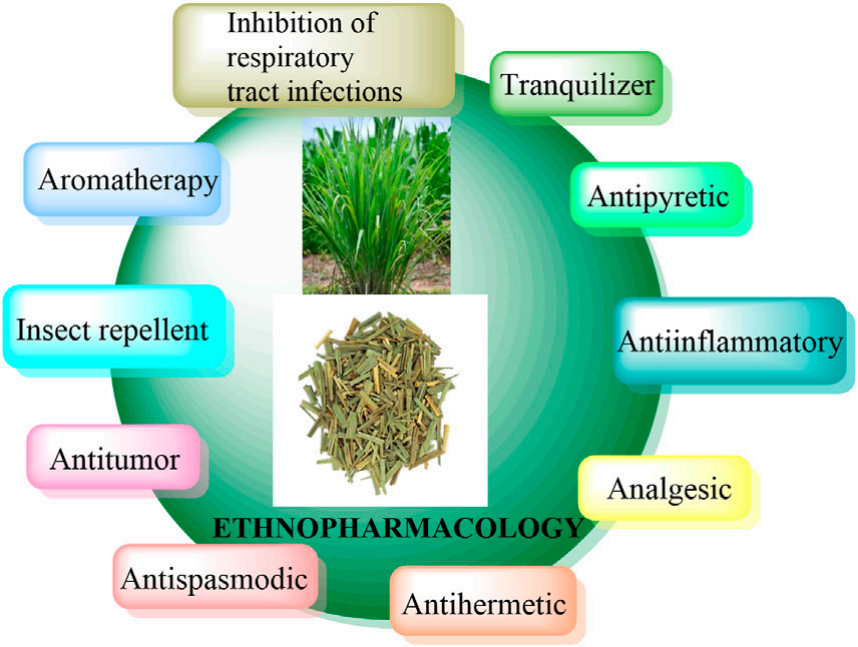
Ethnopharmacological applications of Cymbopogon species.

Cymbopogon species has diverse uses in different countries, contributing to the discovery of traditional medicines and commercial applications. On continents like Asia, South America, and Africa, the Cymbopogon leaves have been traditionally utilized as tea or decoction. Different parts of the Cymbopogon plant embrace key bioactive chemicals which determine the anti-inflammatory, antiseptic, anti-dyspeptic, and anti-fever actions, antispasmodic, analgesic, antipyretic, tranquilizer, anti-hermetic, and diuretic characteristics of the plant (Ademuyiwa et al., 2015; Bayala et al., 2018). In Tanzania, lemongrass tea is utilized as an anti-fever to reduce fever and is used by women to ease dysmenorrhea. The tea is also believed to clean the fallopian tubes, which facilitates easier blood flow. In Singapore, C. citratus is employed to alleviate paronychia, cold and flu symptoms, bug bites, sore and itchy throats, flatulence, indigestion, and cancer prevention (Siew et al., 2014). Interestingly, several countries also choose C. citratus as an insect repellent against mosquitos, house flies, and fleas (Boaduo et al., 2014). In India, C. flexuosus is generally accepted to treat fever, rheumatism, and cancers (Sureshkumar et al., 2017). It has been argued that the aerial parts of *Cymbopogon jwarancusa* (Jones ex Roxb.) Schult (C. jwarancusa) are panacea for respiratory tract infections, while its root decoction has been demonstrated to exert superior roles on dyspepsia, typhoid, and fever in children (Kadir et al., 2014). In Gabon, the crushed leaves of *Cymbopogon densiflorus* (Steud.) Stapf (C. densiflorus) are recognized as a treatment for rheumatism, while its flowerhead is smoked in a pipe as a remedy for bronchial discomfort and asthma in Malawi and Congo. The aerial part and root of *Cymbopogon distans* (Nees ex Steud.) W. Watson (C. distans) are ingeniously utilized as carminatives to prevent heart disease in Pakistan (Ullah et al., 2014). The majority of Cymbopogon essential oils are consumed in aromatherapy because of their therapeutic benefits, which aid in body rejuvenation. It can also be found in a variety of items, including perfumes, local soaps, and candles (Dutta et al., 2016). In several Asian and African countries, the leaf has been shown to have some snake and reptile repellent functions. Figure 3 summarizes some of the ethnopharmacology observed in Cymbopogon species.

## Phytochemistry

**FIGURE 4:**
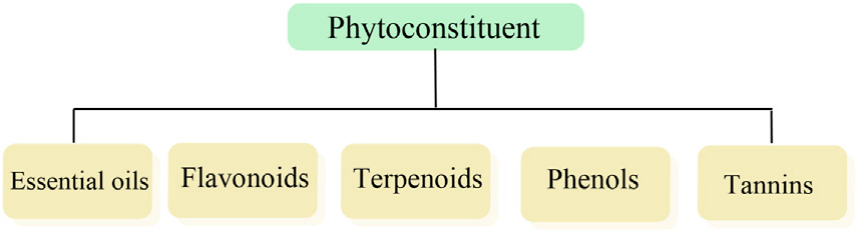
Major phytoconstituents extracted from Cymbopogon species.

**FIGURE 5:**
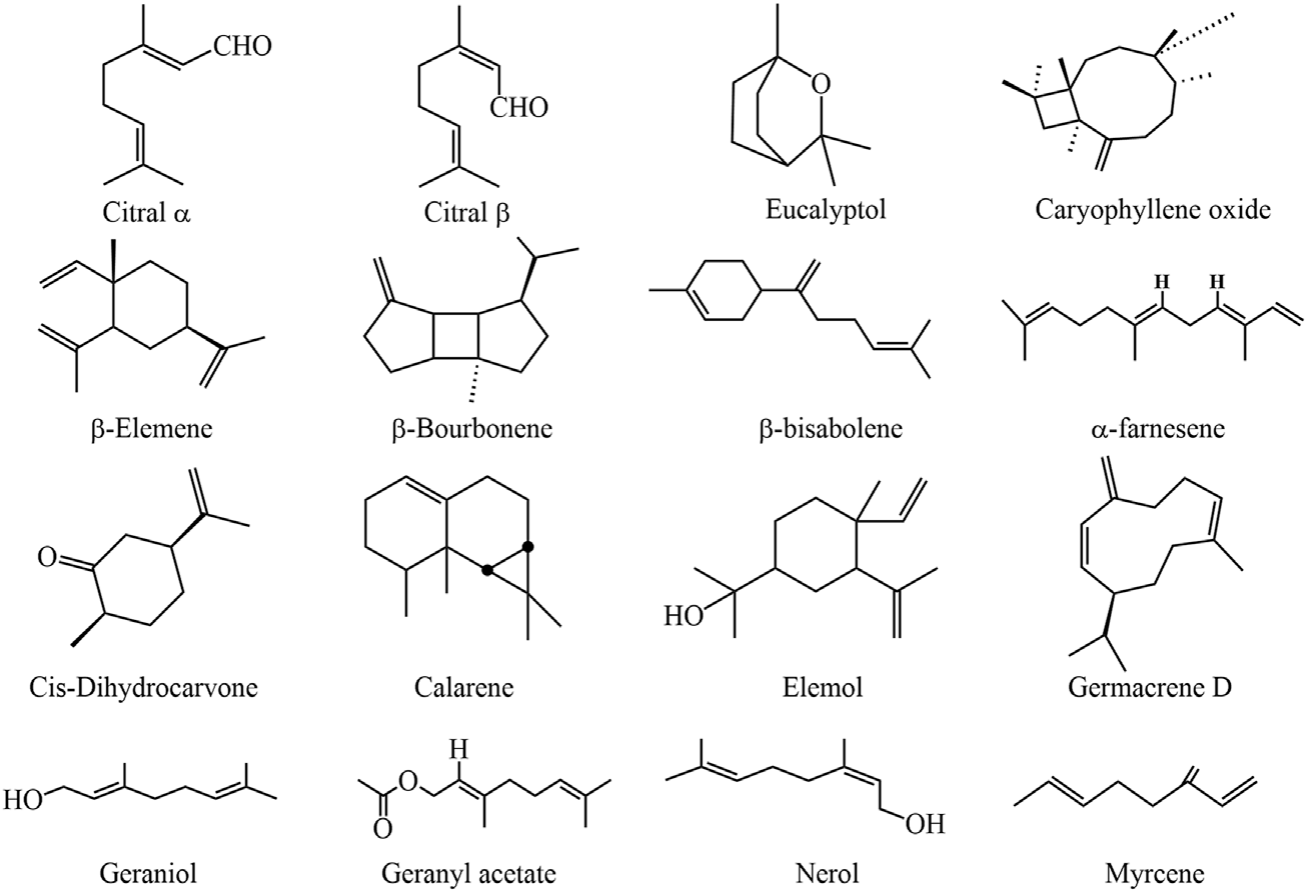
The chemical structures of Cymbopogon essential oils. They include monoterpenes such as citral α, citral β, nerol, geraniol, myrcene, limonene, geranyl acetate and citronellal. Monoterpenoids: Cis-dihydrocarvone, eucalyptol. sesquiterpenes: α-farnesene, β-bisabolene, germacrene D, β-elemene, caryophyllene oxide, calarene. Monoterpene ketone: Piperitone. Sesquiterpenoids: Elemol and β-bourbonene.

The presence of phytochemicals in medicinal herbs may be linked to their therapeutic potential. Many chemical compounds have been isolated from Cymbopogon species, including hydrocarbons, alcohols, ketones, esters, phenols (flavonoids, tannins), volatile and non-volatile terpenoids, acids, carotenoids, and other miscellaneous compounds (Table 2 and Table 3). Among which, essential oils, flavonoids, terpenoids, phenols, and tannins are the major phytoconstituents as shown in Figure 4 (Rahim et al., 2013; Avoseh et al., 2015). The chemical composition of essential oil is mainly composed of monoterpenes, monoterpenoids, sesquiterpenes, sesquiterpenoids (Figure 5), and a few fatty alcohols like 1-Octanol and 4-Nonanol (Piaru et al., 2012; Oladeji et al., 2019).

**TABLE 2:** Phytochemicals isolated from different Cymbopogon species.

Compounds	Species	Phytoconstituent	Extract	Reference
Isoorientin	*Cymbopogon parkeri* Stapf	Flavanoids	Dichloromethane extract	Khanuja et al. (2005)
Tricin	*Cymbopogon parkeri* Stapf, *Cymbopogon bombycinus* (R.Br.) Domin, *Cymbopogon confertiflorus* (Steud.) Stapf, *Cymbopogon procerus* (R.Br.), *Cymbopogon refractus* (R.Br.) A. Camus and *Cymbopogon schoenanthus* (L.) Spreng.	Flavanoids	Dichloromethane extract	Avoseh et al. (2015)
Luteolin	*Cymbopogon citratus* (DC.) Stapf	Flavanoids	Leaves and rhizomes	Padalia et al. (2011)
Cynaroside	*Cymbopogon citratus* (DC.) Stapf	Flavanoids	Leaves and rhizomes	Costa et al. (2016)
Isoscoparin	*Cymbopogon citratus* (DC.) Stapf	Flavanoids	Leaves and rhizomes	Aibinu et al. (2007)
2″-O-rhamnosyl Isoorientin	*Cymbopogon citratus* (DC.) Stapf	Flavanoids	Leaves and rhizomes	Jeong et al. (2009)
Eugenol	*C. ambiguus* A. Camus	Flavanoids	4-phenylpropanoids	Secoy and Smith (1983)
Elemicin	*C. ambiguus* A. Camus	Flavanoids	4-phenylpropanoids	Hilgert (2001)
Eugenol methyl ether	*C. ambiguus* A. Camus	Flavanoids	4-phenylpropanoids	Valdés et al. (2010)
Trans-iso-elemicin	*C. ambiguus* A. Camus	Flavanoids	4-phenylpropanoids	Morton (1981)
Quercetin	*Cymbopogon citratus* (DC.) Stapf	Flavanoids	Aerial parts	Desai and Parikh (2012)
Kaempferol	*Cymbopogon citratus* (DC.) Stapf	Flavanoids	Aerial parts	Noor et al. (2012)
Apigenin	*Cymbopogon citratus* (DC.) Stapf	Flavanoids	Aerial parts	Bagheri et al. (2007)
Catechol	*Cymbopogon citratus* (DC.) Stapf	Flavanoids	Aerial parts	Mahboubi and Kazempour (2012)
Chlorogenic acid	*Cymbopogon citratus* (DC.) Stapf	Flavanoids	Aerial parts	Avoseh et al. (2015)
Caffeic acid	*Cymbopogon citratus* (DC.) Stapf	Flavanoids	Aerial parts	Kepe (2004)
Hydroquinone	*Cymbopogon citratus* (DC.) Stapf	Flavanoids	Aerial parts	Leite et al. (2011)
flavone C-glycosides	*Cymbopogon bombycinus* (R.Br.) Domin, *Cymbopogon confertiflorus* (Steud.) Stapf *Cymbopogon procerus* (R.Br.), *Cymbopogon refractus* (R.Br.) A. Camus and *Cymbopogon schoenanthus* (L.) Spreng.	Flavanoids	Essential oils	Akhila (2009)
Luteferol	*Cymbopogon procerus* (R.Br.) and *Cymbopogon schoenanthus* (L.) Spreng.	Flavanoids	Essential oils	Akhila (2009)
flavonoid sulphate	*Cymbopogon procerus* (R.Br.) and *Cymbopogon schoenanthus* (L.)	Flavanoids	Leaves, essential oils	Akhila (2009)
Apigiferol	*Cymbopogon procerus* (R.Br.) and *Cymbopogon schoenanthus* (L.) Spreng.	Flavanoids	Leaves and essential oils	Akhila (2009)
Umbelliferone	*Cymbopogon parkeri* Stapf	Flavanoids	Leaves and essential oils	Rizk et al. (1995)
P-coumaryl alcohol	*Cymbopogon parkeri* Stapf	Flavanoids	Leaves and essential oils	Rizk et al. (1995)
Quercetin dimethyl ether	*Cymbopogon parkeri* Stapf	Flavanoids	Leaves and essential oils	Rizk et al. (1995)
Prothocyanidins	*Cymbopogon citratus* (DC.) Stapf	Tannins	Leaves	Figueirinha et al. (2008)
Chavicol	*Cymbopogon nardus* (L.) Rendle	Phenols	Leaves and essential oils	Heiba and Rizk (1986)
Elemicine	*Cymbopogon flexuosus* (Nees ex Steud.) and *Cymbopogon nardus* (L.) Rendle	Phenols	Leaves and essential oils	Heiba and Rizk (1986)
Methyl eugenol	*Cymbopogon flexuosus* (Nees ex Steud.), *Cymbopogon nardus* (L.) Rendle and *Cymbopogon distans* (Nees ex Steud.)	Phenols	Leaves and essential oils	Heiba and Rizk (1986)
Phenol	*Cymbopogon coloratus* Stapf	Phenols	Leaves and essential oils	Heiba and Rizk (1986)
Methyl isoeugenol	*Cymbopogon jwarancusa* subsp. olivieri (Boiss.) S. Soenarko *Cymbopogon winterianus* Jowitt ex Bor and *Cymbopogon nardus* (L.) Rendle	Phenols	Leaves and essential oils	Heiba and Rizk (1986)
Cymbodiacetal	*Cymbopogon martinii* Stapf.	bis-monoterpenoid	Leaves	Olivero-Verbel et al. (2010)
Cymbopogone	*Cymbopogon citratus* (DC.) Stapf	Triterpenoids	Leaves	Moreira et al. (2010)
Cymbopogonol	*Cymbopogon citratus* (DC.) Stapf	Triterpenoids	Leaves	Mahabir and Gulliford (1997)
Proximadiol	*Cymbopogon proximus* Stapf	Bicyclic sesquiterpene diol	Leaves	Abdel–Moneim et al. (1969)
Cryptomeridiol	*Cymbopogon parkeri* Stapf	sesquiterpene	Leaves	Locksley et al. (1982)
Eremoligenol	*Cymbopogon parkeri* Stapf	Non-volatile terpenoid	Leaves	Rizk (1986)
Arundoin	*Cymbopogon flexuosus* (Nees ex Steud.)	Triterpene	Leaves	Heiba and Rizk (1986)
Amorphene	*Cymbopogon distans* (Nees ex Steud.)	Hydrocarbon	Leaves and essential oils	Heiba and Rizk (1986)
Bazzanene	*Cymbopogon jwarancusa* subsp. olivieri (Boiss.) S. Soenarko	Hydrocarbon	Leaves and essential oils	Heiba and Rizk (1986)
α- Bergamontene	*Cymbopogon distans* (Nees ex Steud.)	Hydrocarbon	Leaves and essential oils	Heiba and Rizk (1986)
α- Bisabolene	*Cymbopogon flexuosus* (Nees ex Steud.)	Hydrocarbon	Leaves and essential oils	Heiba and Rizk (1986)
β- bisabolene	*Cymbopogon distans* (Nees ex Steud.) Will Watson, *Cymbopogon jwarancusa* subsp. olivieri (Boiss.) S. Soenarko and *Cymbopogon flexuosus* (Nees ex Steud.)	Hydrocarbon	Leaves and essential oils	Heiba and Rizk (1986)
Bourbonene	*Cymbopogon winterianus* Jowitt ex Bor	Hydrocarbon	Leaves and essential oils	Heiba and Rizk (1986)
Camphene	*C. caesius* (Hook and Arn.) Stapf, *Cymbopogon citratus* (DC.) Stapf, *Cymbopogon distans* (Nees ex Steud.), *Cymbopogon martinii* Stapf, *Cymbopogon nardus (*L.) Rendle, *Cymbopogon jwarancusa* subsp. olivieri (Boiss.) S. Soenarko and *Cymbopogon winterianus* Jowitt ex Bor	Hydrocarbon	Leaves and essential oils	Heiba and Rizk (1986)
3-Carene	*Cymbopogon olivieri* (Boiss.) Bor, *Cymbopogon parkeri* Stapf, *Cymbopogon jwarancusa* subsp. olivieri (Boiss.) S. Soenarko, *Cymbopogon flexuosus* (Nees ex Steud.) and *Cymbopogon martinii* Stapf	Hydrocarbon	Leaves and essential oils	Heiba and Rizk (1986)
α- Bisabolol	*Cymbopogon distans* (Nees ex Steud.)	Alcohol	Leaves and essential oils	Heiba and Rizk (1986)
Borneol	*C. caesius* (Hook and Arn.) Stapf, *Cymbopogon citratus* (DC.) Stapf, *Cymbopogon distans* (Nees ex Steud.), *Cymbopogon nardus* (L.) Rendle, *Cymbopogon flexuosus* (Nees ex Steud.) and *Cymbopogon jwarancusa* subsp. olivieri (Boiss.) S. Soenarko	Alcohol	Leaves and essential oils	Heiba and Rizk (1986)
Sesquiterpene alcohols	*C. coloratus* Stapf, *Cymbopogon flexuosus* (Nees ex Steud.), *Cymbopogon distans* (Nees ex Steud.), *Cymbopogon nardus* (L.) Rendle, *Cymbopogon parkeri* Stapf, *Cymbopogon proximus* Stapf and *Cymbopogon schoenanthus* (L.) Spreng.	Alcohol	Leaves and essential oils	Heiba and Rizk (1986)
Limonenediol	*Cymbopogon martinii* Stapf	Alcohol	Leaves and essential oils	Heiba and Rizk (1986)
Benzaldehyde	*Cymbopogon winterianus* Jowitt ex Bor	Aldehyde	Leaves and essential oils	Heiba and Rizk (1986)
β- cardinal	*Cymbopogon winterianus* Jowitt ex Bor	Aldehyde	Leaves and essential oils	Heiba and Rizk (1986)
Octanal	*Cymbopogon distans* (Nees ex Steud.)	Aldehyde	Leaves and essential oils	Heiba and Rizk (1986)
Vanillin	*Cymbopogon winterianus* Jowitt ex Bor	Aldehyde	Leaves and essential oils	Heiba and Rizk (1986)
Decyl aldehyde	*Cymbopogon citratus* (DC.) Stapf	Aldehyde	Leaves and essential oils	Heiba and Rizk (1986)
Carvomenthone	*Cymbopogon distans* (Nees ex Steud.)	Ketone	Leaves and essential oils	Heiba and Rizk (1986)
Cymbopol	*Cymbopogon winterianus* Jowitt ex Bor	Ketone	Leaves and essential oils	Heiba and Rizk (1986)
Pulegone	*Cymbopogon parkeri* Stapf	Ketone	Leaves and essential oils	Heiba and Rizk (1986)
Verbenone	*Cymbopogon jwarancusa* subsp. olivieri (Boiss.) S. Soenarko	Ketone	Leaves and essential oils	Heiba and Rizk (1986)
1-methyl-3-cyclohexanone	*Cymbopogon winterianus* Jowitt ex Bor	Ketone	Leaves and essential oils	Heiba and Rizk (1986)
Geranyl acetate	*Cymbopogon martinii* Stapf, *Cymbopogon nardus* (L.) Rendle	Esters	Leaves and essential oils	Heiba and Rizk (1986)
Geranyl butyrate	*Cymbopogon martinii* Stapf and *Cymbopogon nardus* (L.) Rendle	Esters	Leaves and essential oils	Heiba and Rizk (1986)
Butyl acetate	*Cymbopogon parkeri* Stapf	Esters	Leaves and essential oils	Heiba and Rizk (1986)
Carvyl acetate	*Cymbopogon martinii* Stapf	Esters	Leaves and essential oils	Heiba and Rizk (1986)
Citronellyl formate	*Cymbopogon javanensis* Stapf	Esters	Leaves and essential oils	Heiba and Rizk (1986)
Acetic acid	*Cymbopogon coloratus* Stapf and *Cymbopogon martinii* Stapf	Acids	Leaves and essential oils	Heiba and Rizk (1986)
Butyric acid	*Cymbopogon martinii* Stapf	Acids	Leaves and essential oils	Heiba and Rizk (1986)
Citronellic acid	*Cymbopogon nardus* (L.) Rendle and *Cymbopogon distans* (Nees ex Steud.)	Acids	Leaves and essential oils	Heiba and Rizk (1986)
Perillic acid	*Cymbopogon densiflorus* (Steud.) Stapf	Acids	Leaves and essential oils	Heiba and Rizk (1986)

**TABLE 3:** Major components observed in some Cymbopogon species and their medicinal, traditional and economic uses.

Species	Essential compounds	Uses	Reference
*Cymbopogon bhutanicus* Noltie	——	Perfumery, cosmetics and insect repellant	
*Cymbopogon bombycinus* (R.Br.) Domin	Tricin and flavone C-glycosides	Tea, drinks, food flavoring, antioxidant and skin conditioner	Sharp and Simon (2002)
*C. caesius* (Hook and Arn.) Stapf	Carvone, limonene, perillyl alcohol, citronellol and citronellal	Treating morning sickness in pregnant women and mosquito repellent	Russell et al. (1991); Leistner (2000)
*Cymbopogon citratus* (DC.) Stapf	Camphene, limonene, nonan-4-ol, citronellal, citronellol, neral, geraniol and citral	Antidiarrheal, antibacterial, antifilarial, antifungal, antiinflammatory, antimalarial, hypoglycemic and hypolipidemic effects, and culinary purposes	Adeneye and Agbaje (2007); (Shah et al. (2011)
*Cymbopogon clandestinus* (Nees ex Steud.) Stapf	——	Perfumery and cosmetics	
*Cymbopogon commutatus* Stapf	Limonene	Perfumery and cosmetics	Takaisi-Kikuni et al. (2000)
*Cymbopogon densiflorus* (Steud.) Stapf	trans-p-mentha-2,8-dien-1-ol, cis-p-mentha-2,8-dien-1-ol, trans-p-mentha-1(7),8-dien-2-ol, cis-p-mentha-1(7),8-dien-2-ol and cis-piperitol	Antitumor and antibacterial effects	Pereira et al. (2021); Takaisi-Kikuni et al. (2000)
*Cymbopogon flexuosus* (Nees ex Steud.)	Citral, geranyl acetate, myrcene and citronellol	antiinflammatory effect and cancer chemoprevention	Han and Parker (2017); Sharma et al. (2009)
*Cymbopogon giganteus* Chiov.	trans-p-1(7),8-menthadien-2-ol, cis-p-1(7),8-menthadien-2-ol, trans-p-2,8-menthadien-1-ol, cis-p-2,8-menthadien-1-ol	Antimicrobial, antitrypanosomal, antiplasmodial and antiinflammatory agent	Jirovetz et al. (2007); Kpoviessi et al. (2014); Alitonou et al. (2006)
*Cymbopogon jwarancusa* subsp. olivieri (Boiss.) S. Soenarko	Piperitone-carene, citronellal, p-cymene, geraniol, β-pinene and γ-terpinene	Antipyretic, antirheumatic, antitussive, aromatic, blood purifier, treatment of fever, cough, rheumatism, gout, dyspepsia, cholera and anti-fungi	Heinrich (2003); Bhuyan et al. (2015)
*Cymbopogon proximus* (Hochst. ex A. Rich.) Chiov.	Piperitone, carene, limonene and elemol	Anticonvulsant, anxiolytic, hypotensive, anticonvulsant and antiemetic	(El Tahir and Abdel-Kader (2008)
*Cymbopogon procerus* (R.Br.)	Elemicin and pinene	Tea, culinary purposes and medicinal wash	Thompson and Forster (2021)
*Cymbopogon schoenanthus* (L.) Spreng.	Piperitone, limonene, dihydrocarveol, δ-terpinene, α-terpineol and elemol	Insecticidal effect, antioxidant and ant acetylcholinesterase effect	Ketoh et al. (2006); Khadri et al. (2008)
*Cymbopogon nardus* (L.) Rendle	Citronellal, 2,6-octadienal, caryophyllene, citronellol, limonene and eugenol	Antibacterial and perfumery	Wei and Wee (2013)
*Cymbopogon nervatus* (Hochst.) Chiov.	β-selinene, β-elemene, β-bergamotene, germacrene-D	Molluscidal effect	EL-Kamali et al. (2010)
*Cymbopogon olivieri* (Boiss.) Bor	Piperitone, β-caryophyllene, delta-3-carene, α-eudesmol, α-terpinene and elemol	Antimicrobial effect	Mahboubi and Kazempour (2012)
*Cymbopogon winterianus* Jowitt ex Bor	geraniol, citronellol, citronellal, caryophyllene, citronellyl acetate, elemol, geranyl acetate, linalyl acetate, methyl-iso-eugenol and nerol	Mosquito repellent, molluscidal and antifungal agents	Deletre et al. (2015); Andila et al. (2018)
*Cymbopogon validus* (Stapf) Stapf ex Burtt Davy	artemisia ketone, linalool, northujane, verbenone, naphthalene, δ-cadinene, hedycaryol and α-eudesmol	Anti-inflammatory, anti-rodent, emetic, anti-infective, anti-plasmodic and morning sickness	Rungqu et al. (2016); Akhila (2009); Chagonda et al. (2000)
*Cymbopogon martinii* Stapf	geraniol, citral, citronellol, linalool and geranyl acetate	Preservative, mosquito repellent, anthelmintic and antimicrobial effects	Mishra et al. (2015); Katiki et al. (2011); Prasad et al. (2010)
*Cymbopogon pendulus* Stapf	citral-a, citral-b, geranyl acetate, β-caryophyllene, elemol, geraniol and linalool	Antifungal effect	Pandey et al. (1996)
*Cymbopogon distans* (Nees ex Steud.)	Terpineol, piperitone, geraniol, limonene and methyl eugenol	Anti-inflammatory, cough, common cold, asthma, chronic bronchitis, antibacterial and antifungal effects	Zhang et al. (2011)

### Cymbopogon terpenoids

a) Non-volatile terpenoids: C. martinii produces cymbodiacetal (Olivero-Verbel et al., 2010), a new bis-monoterpenoid, whereas C. citratus leaves yield the triterpenoids cymbopogone (Moreira et al., 2010) and cymbopogonol (Mahabir and Gulliford 1997), both of which manifest as non-volatile. b) Volatile terpenoids: Volatile terpenoids are abundant in the Cymbopogon genus of different species, some of which include citral, geraniol, citronellol, piperitone, and elemin.

### Flavonoids

Antioxidant characteristics were found in this family of compounds. Isoorientin and tricin were extracted from a dichloromethane extract and the whole plant of C. parkeri (Rizk et al., 1995), and their muscular relaxation activity had been determined (Rizk et al., 1986). Other flavonoid compounds like cynaroside, luteolin, isoscoparin, and 20-O-rhamnosyl isoorientin were isolated from the leaves and rhizomes of C. citrates, while apigenin, kaempferol, caffeic acid, catechol, quercetin, chlorogenic acid, hydroquinone, and elemicin were identified from the aerial parts of C. citratus (Asif and Khodadadi, 2013; Roriz et al., 2014).

### Tannins

C. citratus is a Cymbopogon specie whose tannin content is heavily exploited. The species from Portugal and fractionated extracts contained about 10 mg of hydrolysable tannins (prothocyanidins) (Figueirinha et al., 2008), whereas C. citratus from Nigeria embraced about 0.6 percent tannins. Some studies have also proven the content of condensed tannins in C. nardus (Gebashe et al., 2020).

### Phenol

In a particular study, total soluble phenolic content was obtained in methanolic root extracts of C. nardus, wherein it was shown that its content ranged from 4.2 to 30.9 mg GAE/g DW (milligrams of gallic acid equivalents per g). p-coumaric, ferulic, and chlorogenic acids are some of the phenolic acids isolated from the roots of C. nardus (Gebashe et al., 2020).

### Essential oils

Cymbopogon essential oils are widely used in the fragrance, cosmetic, food, and flavor industries. Pain alleviation and blood sugar regulation are some of the biological effects of the oil (Dikshit 1984). The famous Cymbopogon essential oils include lemongrass oils (obtained from *C. flexuosus*, *C. citratus,* and *C. pendulus*), citronella oils (obtained from *C. winterianus* and C. nardus), palmarosa and ginger grass oils (obtained from C. martinii) (Husain 1994). Other Cymbopogon species known to produce essential oils are *C. schoenanthus* (camel grass), *C. caesius* (inchi/kachi grass), *C. afronardus*, *C. clandestinus*, *C. coloratus*, *C. exaltatus*, *C. goeringii*, *C. giganteus*, *C. jwarancusa*, *C. polyneuros*, *C. procerus, C. proximus, C. rectus, C. sennaarensis, C. stipulatus,* and *C. virgatus* (Akhila 2009). Geraniol and citral are two major constituents of the essential oil that, due to their specific rose and lemon-like aromas, are the preferred raw material for the commercial production of flavors, cosmetics, and fragrances in soaps and detergents (Ganjewala and Luthra 2010). Citral-containing species includes *C. flexuosus*, *C. martinii*, *C. citratus*, *C. pendulus*, and *Cymbopogon schoenanthus*. While geraniol-containing species covers in *C. flexuosus*, *C. jwarancusa*, *C. martinii*, *C. citratus*, *C. pendulus*, *C. winterianus*, *C. nardus*, *C. caesius*, *C. coloratus*, *C. parkeri*, etc. Table 4 summarizes some of the essential oils found in Cymbopogon species.

**TABLE 4:** The essential oils found in some Cymbopogon species.

Essential oil	Species	Percentage	Reference
α-thujene	*C. caesius* (Hook and Arn.) Stapf	0.57	Kanjilal et al. (1995)
α-pinene	*Cymbopogon pendulus* Stapf, *C. caesius* (Hook and Arn.) Stapf, *Cymbopogon jwarancusa* subsp. olivieri (Boiss.) S. Soenarko, *Cymbopogon javanensis* Stapf, *Cymbopogon distans* (Nees ex Steud.), *Cymbopogon excavatus* Stapf, *Cymbopogon flexuosus* (Nees ex Steud.) and *Cymbopogon proximus* (Hochst. ex A. Rich.) Chiov	6.10, 0.99 and 0.050	Saeed et al. (1978); Kanjilal et al. (1995)
α-Terpineol	*Cymbopogon martinii* Stapf,	7.19	Kalagatur et al. (2020)
β-ocimene	*Cymbopogon martinii* Stapf,	0.66	Kaur et al. (2019)
β-caryophyllene	*Cymbopogon martinii* Stapf and *Cymbopogon citratus* (DC.) Stapf	1.36 and 3.91	Almeida et al. (2018); Kaur et al. (2019)
Bornyl acetate	*C. caesius* (Hook and Arn.) Stapf, *Cymbopogon distans* (Nees ex Steud.), *Cymbopogon parkeri* Stapf and *Cymbopogon flexuosus* (Nees ex Steud.)	0.60 and 4.80	Kanjilal et al. (1995); Akhila (2009)
β-Caryophyllene	*Cymbopogon martinii* Stapf, *Cymbopogon citratus* (DC.) Stapf, *Cymbopogon caesius* (Hook. & Arn.) Stapf, *C. jwarancusa* subsp. olivieri (Boiss.) S. Soenarko, *Cymbopogon nardus* (L.) Rendle, *Cymbopogon distans* (Nees ex Steud.) and *Cymbopogon flexuosus* (Nees ex Steud.)	1.36, 3.91, 0.85 and 1.20	Saeed et al. (1978); Kanjilal et al. (1995); Zahra et al. (2020)
Carveol	*Cymbopogon densiflorus* (Steud.) Stapf, *Cymbopogon commutatus* Stapf, *Cymbopogon excavatus* Stapf, *Cymbopogon martinii* Stapf, *Cymbopogon nervatus* (Hochst.) Chiov and *Cymbopogon proximus* (Hochst. ex A. Rich.) Chiov	2.37	Seibert et al. (2018)
Neryl acetate	*Cymbopogon parkeri* Stapf	3.78	Rizk et al. (1983)
Citronellyl valerate	*Cymbopogon javanensis* Stapf	Traces	Heiba and Rizk (1986)
Citral α	*Cymbopogon citratus* (DC.) Stapf, *Cymbopogon winterianus* Jowitt ex Bor and *Cymbopogon flexuosus* (Nees ex Steud.)	40.80, 8.05 and 42.40	Quintans-Júnior et al. (2008); Shah et al. (2011); Bharti et al. (2013)
Citral β	*Cymbopogon citratus* (DC.) Stapf, *Cymbopogon giganteus* Chiov and *Cymbopogon flexuosus* (Nees ex Steud.)	32.00, 26.50 and 33.31	Kasali et al. (2001); Matasyoh et al. (2011); Costa et al. (2013)
Citronellal	*Cymbopogon nardus* (L.) Rendle and *Cymbopogon winterianus* Jowitt ex Bor C. martini	27.52, 16.33 and 2.37	Solanki et al. (2019); Cunha et al. (2020); Kalagatur et al. (2020)
Citronellol	*Cymbopogon nardus* (L.) Rendle, *Cymbopogon winterianus* Jowitt ex Bor	25.00, 14.26	Solanki et al. (2019); Cunha et al. (2020)
Caryophyllene oxide	*Cymbopogon martinii* Stapf	0.97	Kaur et al. (2019)
Eudesmol	*Cymbopogon proximus* Stapf	6.60	El Tahir and Abdel-Kader (2008)
Elemol	*Cymbopogon winterianus* Jowitt ex Bor	15.58	Solanki et al. (2019)
Fernesol acetate	*Cymbopogon martinii* Stapf,	0.68	Kaur et al. (2019)
Furfural	*Cymbopogon citratus* (DC.) Stapf, *Cymbopogon winterianus* Jowitt ex Bor	Traces	Akhila (2009)
Farnesol	*Cymbopogon martinii* Stapf	9.43	Zahra et al. (2020)
Geranyl butanoate	*Cymbopogon parkeri* Stapf	0.69	Rizk et al. (1983)
Geranyl hexanoate	*Cymbopogon martinii* Stapf and *Cymbopogon parkeri* Stapf	8.0 and 1.09	Kaur et al. (2019)
Geraniol	*Cymbopogon citratus* (DC.) Stapf and *Cymbopogon martinii* Stapf	2.66 and 70.26	Almeida et al. (2018); Jummes et al. (2020)
Geranyl acetate	*Cymbopogon flexuosus* (Nees ex Steud.) and *C. caesius* (Hook and Arn.) Stapf	12.0 and 3.36	Kanjilal et al. (1995); Chowdhury et al. (2010)
Geranyl proprionate	*Cymbopogon martinii* Stapf	6.40	Kalagatur et al. (2020)
β-bisabolene	*Cymbopogon distans* (Nees ex Steud.)	5.4	Akhila (2009)
Limonene	*Cymbopogon giganteus* Chiov, *Cymbopogon nardus* (L.) Rendle, *Cymbopogon proximus* Stapf, *C. caesius* (Hook and Arn.) Stapf, *Cymbopogon martinii* Stapf, *Cymbopogon distans* (Nees ex Steud.), *Cymbopogon flexuosus* (Nees ex Steud.), *Cymbopogon densiflorus* (Steud.) Stapf, *Cymbopogon jwarancusa* subsp. olivieri (Boiss.) S. Soenarko, *Cymbopogon winterianus* Jowitt ex Bor, *Cymbopogon excavatus* Stapf, *Cymbopogon nervatus* (Hochst.) Chiov, *Cymbopogon olivieri* (Boiss.) Bor, *Cymbopogon parkeri* Stapf and *Cymbopogon sennaarensis* Chiov	19.3, 3.5, 3.9, 7.26, 20, 12.08, 2.4, 52.1, 0.08 and 3.91	Saeed et al. (1978); Kanjilal et al. (1995); Akhila (2009); Bayala et al. (2018); Cunha et al. (2020)
Linalool	*Cymbopogon martinii* Stapf and *Cymbopogon winterianus* Jowitt ex Bor	1.16 and 16.95	Kaur et al. (2019); Solanki et al. (2019)
Mentha-1(7),8-dien-2 ol cis	*Cymbopogon giganteus* Chiov.	17.34	Bayala et al. (2018)
Myrcene	*Cymbopogon citratus* (DC.) Stapf	18.0	Chisowa et al. (1998)
Nerol	*Cymbopogon nardus* (L.) Rendle	21.89	Cunha et al. (2020)
Neryl butanoate	*Cymbopogon parkeri* Stapf	0.69	Rizk et al. (1983)
p-cymene	*Cymbopogon distans* (Nees ex Steud.)	5.1	Akhila (2009)
Camphene	*Cymbopogon jwarancusa* subsp. olivieri (Boiss.) S. Soenarko	0.10	Saeed et al. (1978)
Piperitenone	*Cymbopogon khasianus* (Hack.) Stapf ex Bor and *Cymbopogon olivieri* (Boiss.) Bor	Traces	Akhila (2009)
Piperitone	*Cymbopogon olivieri* (Boiss.) Bor *Cymbopogon parkeri* Stapf and *Cymbopogon proximus* (Hochst. ex A. Rich.) Chiov.	72.8, 80.8 and 59.1	Avoseh et al. (2015)
Perillyl alcohol	*Cymbopogon martinii* Stapf	15	Akhila (2009)
Perillaldehyde	*C. caesius* (Hook and Arn.) Stapf	4.01	Kanjilal et al. (1995)
Thymol	*Cymbopogon martinii* Stapf	6.19	Kalagatur et al. (2020)
Trans-geraniol	*Cymbopogon martinii* Stapf	66.9	Kaur et al. (2019)
Trans-nerolidol	*Cymbopogon martinii* Stapf	1.38	Kaur et al. (2019)
Trans-Mentha-2,8-diene-para-ol 1	*Cymbopogon giganteus* Chiov.	13.91	Bayala et al. (2018)

### Mineral contents

In a study performed on Cymbopogon citratus, some of the reported essential mineral constituents were potassium (K), sodium (Na), calcium (Ca), magnesium (Mg), manganese (Mn), iron (Fe), zinc (Zn), phytate and phosphorus (P) (Boukhatem et al., 2014). Other minerals that can be found in C. citratus include chromium (Cr), nickel (Ni), copper (Cu), arsenic (As), cadmium (Cd), and lead (Pb) (Anal 2014). Essential minerals found in Cymbopogon schonenanthus include Ca, P, K, Mg, Cu, Zn, Mn and cobalt (Co.) (Alameen 2020). The percentage of mineral content was calculated at a concentration of (mg/100 g) while in other reports the content was converted from (parts per million) ppm to percentage (as shown in Table 5 **)**.

**TABLE 5:** Essential mineral components found in Cymbopogon species.

Species	Minerals	Percentage	Reference
*Cymbopogon schoenanthus (L.)* Spreng	Ca	0.49	Alameen (2020)
	P	0.032	
	K	0.48	
	Mg	0.022	
	Cu	0.0023	
	Zn	0.00035	
	Mn	2.70	
	Co.	0.0000023	
*Cymbopogon citratus* (DC.) Stapf	Na	0.74	Boukhatem et al. (2014); Anal (2014)
	K	2.12	
	Ca	0.36	
	Mg	0.15	
	P	0.07	
	S	0.19	
	Fe	0.013	
	Mn	0.016	
	Zn	35.51	
	Cu	0.0057	

## Pharmacology

**FIGURE 6:**
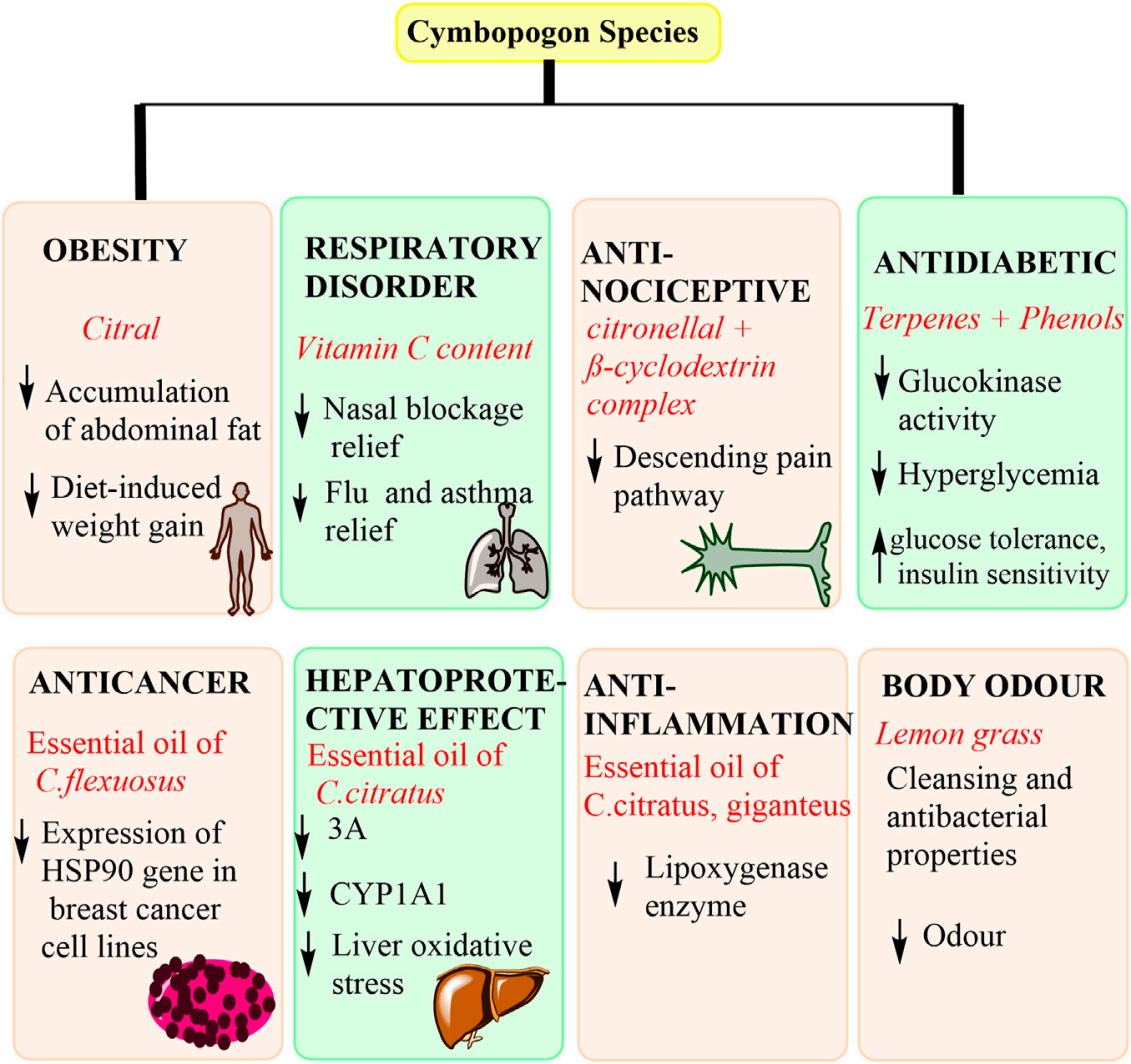
The involved mechanisms of action observed in Cymbopogon species for disease management.

Most of the pharmacological investigations have been conducted based on the components present. These components have led to the discovery of different pharmacological effects, which are proved by the mechanism of action discovered. Figure 6 highlights some of the mechanisms of action observed in Cymbopogon species.

### Antiprotozoal effect

Citral is the major component of *C. citratus* and *C. flexuosus* essential oils. According to some findings, myrcene and citral have been discovered to have an antileishmanial effect on different species like Leishmania infantum, Leishmania tropica, and Leishmania major (Ganjewala et al., 2012). Citral, on the other hand, has been shown to exert anti-trypanosoma cruzi activity (Cardoso and Soares 2010) and to inhibit the anatomic and ultrastructural changes of Leishmania amazonensis without cytotoxicity (Santin et al., 2009). In other studies, Entamoeba histolytica was shown to be active in broth culture from cymbopogon essential oil (Dutta et al., 2016).

### Anti-bacterial and antifungal effect

C. citratus has been used to isolate, characterize, and analyze essential oils like citral (geranial) and citral (neral), which have been shown to be antibacterial chemicals that are active against both gram-positive and gram-negative microorganisms (Soares et al., 2013). Bacterial infections like meningitis, pneumonia, impetigo, cellulitis, folliculitis and food poisoning have been treated traditionally by this herb (Ntulume et al., 2019). C. jwarancusa oil can distinctly inhibit the growth of various bacteria like *Klebsiella pneumoniae*, Citrobacter, *Proteus mirabilis*, *Salmonella* enteric sertyphi, and *Shigella* flexneri species (Prasad et al., 2014). Marvelously, myrcene has been shown to have low antibacterial activity but is very active when combined with other essential oils (Orafidiya 1993; Wannissorn et al., 2005). While the essential oil of C. martinii is reported to possess magnificent antibacterial activity against *Staphylococcus aureus*, S. pyagens, *E. coli*, and Corynebacterium ovis (Chouhan et al., 2017).

Cymbopogon oil has been affirmed to be one of the most effective anti-dermatophytes medications. Examples of dermatophytes treated by this herb include Trichophyton mentagrophytes, T. rubrum, Epidermophyton floccosum, and Microsporum gypseum (Wannissorn et al., 1996). Cymbopogon essential oils have also been corroborated to possess prominent resistance to pathogenic fungal stimulus that cause problems with mycotoxins released during grain storage and restrain filamentous fungus development by yeast cells, which has been reported to have antagonistic and synergistic effects on food storage (Nguefack et al., 2012; Kpoviessi et al., 2014). Coincidently, *C. jwarancusa* is proven to be extremely efficient against Fusarium oxyporium sp–lini (Dutta, Munda et al., 2016). Conformably, *C. citratus* can suppress fungal infections like athlete’s foot, ringworm, jock itch, yeast infections, and keratinophilic fungi (Abe et al., 2003).

### Anti-cancer effect

In different studies, the essential oils of C. densiflorus and C. flexuosus were analyzed to see if they had antitumor effects. Evidence suggested that C. flexuosus oil triggered apoptosis in human leukemia cell lines (HL-60 cells) *in vitro* and a murine sarcoma inoculated with S-180 tumor cells *in vivo*. Amazedly, the greatest effect was seen on the human cancer lines of colon, liver, cervix, and neuroblastoma. The oil treatment of *C. densiflorus* is suggested to have antitumor effects on TP53 wild-type and mutated bladder cancer cells. These results indicate that C. flexuosus and C. densiflorus oils could be a reasonable alternative for novel antineoplastic drugs (Sharma et al., 2009; Pereira et al., 2021).

### Anti-inflammatory effect

Chronic inflammation is a major worldwide health problem that has been associated with life-threatening conditions like cancer (Colotta et al., 2009). Cymbopogon’s ethnopharmacological studies have confirmed citral to have anti-inflammatory properties. The essential oil of C. flexuosus, which has high citral content, has been announced to substantially reduce numerous inflammatory biomarkers in human skin cells and conspicuously subdue chemical agents-induced skin inflammation both topically and orally in a mouse model (Han and Parker 2017). Currently, it has been widely used as an ingredient in lotions and ointments to treat topical inflammation (de Cassis da Silveira e Sa et al., 2013; Boukhatem et al., 2014).

### Antimalarial and antitrypanosomal effect

The essential oils of *C. giganteus*, *C. citratus*, *C. nardus*, and *C. schoenanthus* have been shown to have an effect against Trypanosoma brucei brucei and Plasmodium falciparum. Some secondary metabolites like citral (3, 7-dimethyl-2, 6-octadienal), myrcene, and citronellal were referred to as antimalarial compounds when compared to chloroquine, as they were found to decrease Plasmodium’s growth by 86.6 percent (Melariri 2010; Arrey Tarkang et al., 2014; Kpoviessi et al., 2014). While, elemicin was declared to be present in 53.7 percent of *C. pendulus* essential oil. This compound is an essential starting material for the synthesis of the antimalarial drug trimethoxy prim. In another study, the ethanol extracts of C. citratus essential oil were shown to ameliorate the antioxidant state of oxidative stress-related malaria effects (Tchoumbougnang et al., 2005; Akono Ntonga et al., 2014).

### Antidiabetic effect

*C. nardus* is indicated to be efficient in maintaining blood glucose levels despite a lack of details on its mode of action (Widiputri et al., 2019). Studies have revealed the antidiabetic potency of *C. citratus* at dosage rates of 400 and 800 mg, by decreasing the levels of insulin (*p* < 0.001), glucose (*p* < 0.001) and triglycerides (*p* < 0.001) (Bharti et al., 2013). Another report on the effect of lemongrass tea (mainly containing phenolics and terpenes) in a type 2 diabetes rat model suggested that the inhibition of glucokinase activity attributed to its antidiabetic effect, which resulted in enhancement of hyperglycemia, glucose tolerance ability, insulin sensitivity, T-cell functions, and dyslipidemia (Garba et al., 2020).

### Anti-obesity and antihypertensive effect

Cymbopogon leaves have been commonly consumed as tea to manage glucose, lipid, and fat levels in the blood serum, which may help to prevent obesity and hypertension (Shah et al., 2011). An experiment conducted with an aqueous extract of *C. citratus* showed a decrease in fasting plasma, glucose, total cholesterol, triglycerides, low-density lipoproteins, and very low-density lipoprotein while raising the plasma high-density lipoprotein level of rats with no effect on the plasma triglyceride levels (Adeneye and Agbaje 2007). Similarly, C. nardus could also console obesity by downregulating adipogenic and lipogenic genes (Ruangaram and Kato 2022). The evidence also suggested that Cymbopogon could reduce blood pressure and maintain blood glucose by secreting insulin (hyperinsulinemia) (Shimono et al., 2010).

### Antinociceptive effect

Cymbopogon has long been used as a traditional medicine to relieve pain and anxiety in living beings (Ademuyiwa et al., 2015). Citronellal, a monoterpene observed in several Cymbopogon species, demonstrated the inhibition of descending pain pathways with anti-hyperalgesic actions that lasted longer when forming a β-cyclodextrin complex (Santos et al., 2016). While the essential leaf oil of *C. winterianus* exhibits antinociceptive effects through the inhibition of prostaglandin synthesis (Leite et al., 2010).

### Insecticidal activity

Pathogens and insects have been controlled using essential oils from different Cymbopogon species, for example, piperitone from C. schoenanthus is proven to be a strong repellant against Crematogaster spp (Bowers et al., 1993) and Callosobruchus maculatus (Ketoh et al., 2006). The essential oils of *C. citratus, C. nardus*, and *C. martini* are reported to be extremely efficient against anopheline mosquitos, Anopheles culicifacies, Anopheles quinquefasciatus (Ansari and Razdan 1995), and Aedes aegypt by the essential oil of *C. flexuosus* (Osmani and Sighamony 1980).

### Anti-HIV effect

In HIV/AIDS patients, citronella oil extracted from *C. citratus* leaves was reported to heal oral thrush caused by *Candida* albicans in 1–5 days (Wright et al., 2009).

### Perfumery

Most Cymbopogon species could produce a lot of essential oils that are aromatic, hence their extensive use in perfumery and the fragrance industry. Known as natural fragrance, Java citronella oil is used for the extraction of aromatic isolates, which has been added to several products due to their basic aromas. In East Indian perfumery, lemongrass oil from *C. pendulus* is the primary source. For *C. winterianus* oil, it provides scent and good odor to low-cost items such as sprays, disinfectants, and polishes. Palmarosa oil from *C. martinii* is extensively used in soaps more than any other oil due to its rose-like prominent and lasting odor, which can also be found in some mouth fresheners (Guenther and Althausen, 1948; Opdyke 1975). Other oils that can be used in perfuming soaps and scenting cosmetics include the essential oil of *C. caesius*, ginger grass oil, Palmarosa oil, *C. winterianus* oil, Java citronella oil, etc.

## Toxicology

Generally, Cymbopogon is safe when consumed orally, topically, or used as aromatherapy. However, there are some factors, like heavy metal contamination from the soil, interaction of Cymbopogon with some drugs, and wrong consumption that can cause an undesirable effect (Ekpenyong et al., 2015a). According to some studies, citronella oil derived from the species C. afronardus, C. nardus, C. validus, and C. winterianus, is one of the ingredients in insect repellents and is applied topically to prevent mosquito bites. However, some adverse effects have aroused public concern. This includes skin reactions or irritation in some people, lung damage prior to its inhalation, and some reports of child poisoning after ingesting citronella oil-contained insect repellent (RxList 2021a, November 6). Other negative effects documented prior to the use of lemon grass include skin allergies when used directly on the skin, toxic alveolitis when inhaled, an elevation in amylase, and bilirubin (RxList 2021b, February 9). Relevant findings suggest that toxicological biomarkers and genotoxicity of *C. citratus* essential oil have a low toxicity level but are suitable and safe in long-term treatment at doses up to 100 mg/kg (Bidinotto et al., 2011). Although the United States has classified lemongrass as relatively safe., it is not recommended for use during pregnancy and breast-feeding, considering its stimulation to uterine and menstrual flow (Nimenibo-Uadia and Nwosu, 2020).

## Quality control

**FIGURE 7:**
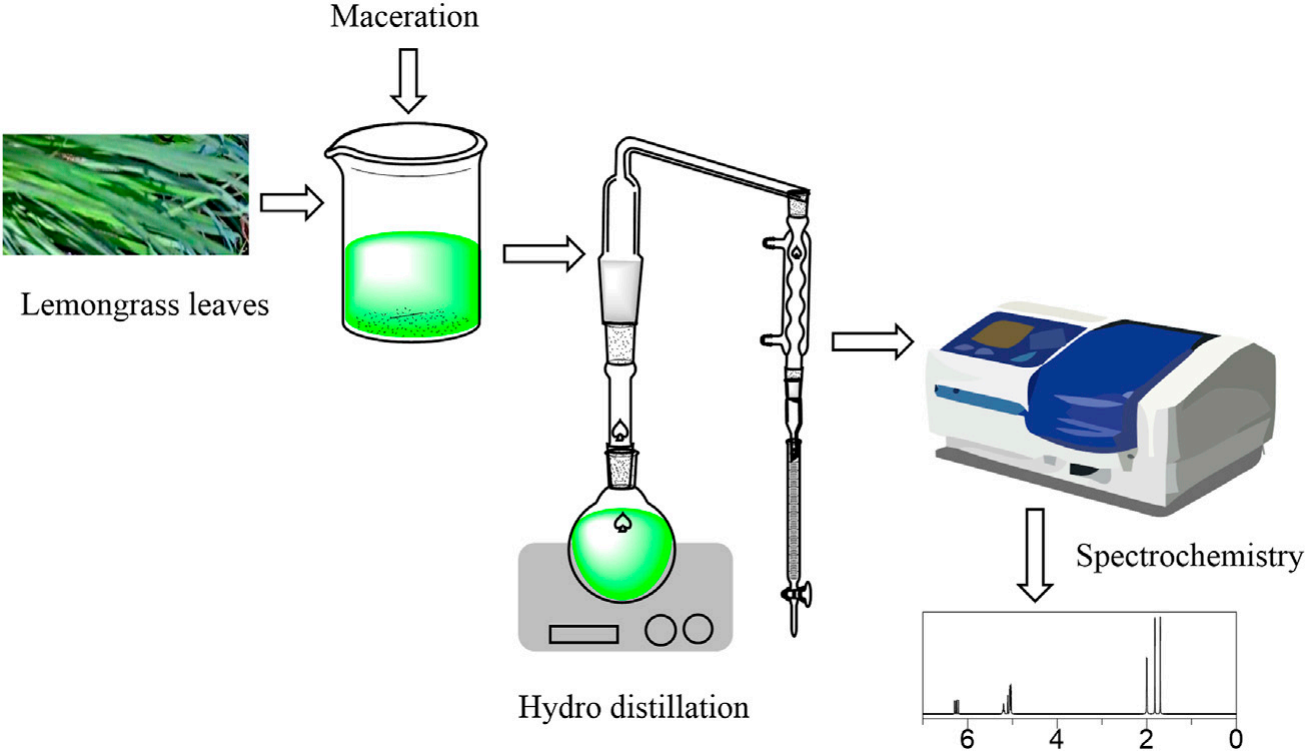
Plant collection, extraction, isolation, and analysis of constituents in Cymbopogon genus.

Quality control is important for medicinal herbs as it helps in identifying the herb, discovering the active and minimal components, and becoming acquainted with the extraction, separation, or isolation process of the herb (Wang et al., 2019). Figure 7 summarizes this process of Cymbopogon genus. This was conducive to assure the safety of the herb, its ethnopharmacology, and its pharmacological effects.

### Plant collection

Fresh plant leaves are obtained and then washed rigorously with either distilled water or normal saline. The leaves are then ground into fine powder with the aid of an electric blender and then stored at room temperature.

### Extraction process

Via the maceration technique, several grams of powdered plant leaves are extracted with a litre of methanol in a stopped bottle overnight while being stirred periodically. And the sample is sieved and then filtered using filter paper. After that, several grams of plant leaves are simmered in a conical flask with 500 ml of double-distilled water for 1 h. The decoction is cooled for 3 h and then filtered using a piece of clean, sterile, white cotton cloth. The extract/filtrate is then concentrated to interesting concentrations using a rotary evaporator at a specific temperature, and stored in an airtight container and kept in the freezer until further use. The concentrate can then be used to prepare different concentrations (mg/ml) of the extract using distilled water (Adeneye and Agbaje, 2007; Elekofehinti et al., 2020).

### Isolation process

Isolation of essential oils is done via hydrodistillation, where several grams of plant leaves are steam distilled for 3 h in a modified Clevenger-type apparatus. The essential oils are stored in a sealed vial at a specific temperature and the yields are calculated referring to the following formula: Yield (%) = (Oil (ml))/(Plant (g)) × 100 (Kpoviessi et al., 2014; Tavares et al., 2015; Mendes Hacke et al., 2020). The yields are based on the volume of water, the size of the leaves, and the extraction time (Thakker et al., 2016).

### Physicochemical and spectroscopic techniques

The basic physical and chemical properties of a substance are known as physicochemical properties. This includes color, boiling temperature, refraction index, density, volatility, water solubility, and flammability. According to the research done on Cymbopogon citratus, an Atago ND R5000 refractometer (Osaka, Japan) was used to measure the refractive index of oil, according to the 921.08 AOAC (2000) method. The density was evaluated according to the 985.19 AOAC (2000) method using a 2 ml pycnometer. The density and refractive index are in the range (ρ = 0.848–0.949 g/ml; IR = 1.332–1.482). A yellow hue was detected using a Color Guard 05 colorimeter in transmittance mode when measuring the color parameters (Paviani et al., 2006; Essien et al., 2008; Tovar et al., 2011; del Carmen Vázquez-Briones et al., 2015). Spectroscopic techniques are precise analytical methods used to discover the structures of atoms, molecules, and unknown chemical compositions in Cymbopogon genus. Some of the techniques used to discover Cymbopogon essential oils and constituents include gas chromatography-mass spectrometry, fourier transform infrared spectrometry, nuclear magnetic resonance, differential scanning calorimetry, and scanning electron microscopy (Fortuna et al., 2011; del Carmen Vázquez-Briones et al., 2015; da Silva Martins et al., 2021).

## Discussion

The use of traditional medicines is widespread and dates back a long time before the increase in technology and knowledge that we have now (Wang et al., 2019 and, 2022). Cymbopogon species can also be termed as medicinal plants due to their many reported medical benefits, which mostly ascribe to their essential oils and the whole parts of the plant in general. Most Cymbopogon species are declared relatively safe to consume, except for some like *C. winterianus*, *C. schoenanthus*, *C. pendulus*, and *obtectus*, whose edible uses are unknown but are known to be used for medicinal purposes, perfumery products, cosmetics and household insecticides. Traditionally, the use of these species varies from simply being consumed as tea, drinks, or food flavoring. Medically, they have been argued to exhibit: a) anti-inflammatory, antiseptic, anti-dyspeptic, and anti-fever actions; b) antispasmodic, analgesic, antipyretic, tranquilizer, anti-hermetic, and diuretic anti-fever; c) ease dysmenorrhea; d) detoxication effects on the kidney and liver. The dosage used traditionally is not standard nor scientifically approved. However, it is believed in most countries that one tea cup of lemongrass daily can strengthen individual’s immunity. Clinically, citral, the main component in lemongrass, together with its other components, is thought to have anticancer ability and reduce the risk of various developing cancers by attacking the cancer cells or by boosting the immunity system. On the other hand, some cancer patients are given lemongrass tea after going through rounds of chemotherapy or radiation for relief. A study manifested that C. citratus tea can help get rid of gastric ulcers or even reduce the risk of gastric ulcers obtained from synthetic drugs like aspirin (Fernandes et al., 2012). Evidence also documented that drinking lemon grass tea every day for a month boosted the red blood cell count by increasing the amount of hemoglobin concentration, which was attributed to its superior antioxidant properties (Ekpenyong et al., 2015b).

Furthermore, many investigations have been dedicated to exploring the phytochemistry, phytotherapy and pharmacology of Cymbopogon species. In one of the studies, it has been reported that a dichloromethane extract of C. ambiguous was demonstrated to have antiplatelet activity, which is believed to be highly because of its eugenol content (Uddin et al., 2011). Another study substantiated that the methanolic extract of C. citratus can achieve a dose-dependent relaxant activity through endothelial vasoconstriction via the nitric oxide pathway (Pereira et al., 2013). As to adverse effects, it is generally safe to consume some of the Cymbopogon species, but several adverse effects have been reported by oral or topical administration. Adverse effects observed orally include fatigue, dry mouth, excessive urination, and vertigo. Noteworthily, lemongrass essential oil might harm the mucous membranes in the stomach and liver when excessive and unrestrained use. When applied topically, skin reactions or irritation can be observed. It is also advisable to avoid taking it with drugs that are glutathione-S-transferase substrates and cytochrome p450 substrates to prevent any risk of increased side effects of these drugs. Moreover, pregnant and breast-feeding mothers can also avoid consuming lemongrass tea, citronella oil and its related products, so as to prevent its effects on infant development (Memorial Sloan Kettering Cancer Center 2020, February 14).

## Conclusion and future prospects

Cymbopogon species are very fascinating, diverse, and rich in every sense, from their simple traditional uses like culinary and tea. Of which some of the species like C. nardus are not edible due to their unpleasant nature, while some remain unknown as to whether they are edible or not. To their medicinal uses, the robust pharmacological activity is mainly attributed to their richness in essential oils, such as monoterpenes, monoterpenoids, sesquiterpenes, sesquiterpenoids, and certain fatty alcohols. This genus is widespread throughout the world, with species like C. giganteus and C. densiflorus found in tropical Africa, C. nardus in eastern Africa, C. caesius in southern and eastern Africa, C. martinii, C. flexuosus, C. distans, and C. coloratus in eastern Asia, among others, all of which have unique medicinal and commercial benefits. Lastly, more research is needed to improve the ones that are known and to reveal those with little knowledge. Researchers can come up with more essential products that can be made using natural ingredients to replace those manufactured with harsh chemicals, which cause a lot of side effects and negative health factors. In addition, the drug dosage and mode of drugs traditionally given to people should be effectively tested, and this will aid in the increase of certified medicinal drugs and enhanced pharmacological effects in the future. Meanwhile, the exact clinical efficacy and potential organ toxicity of some species or active ingredients also need to be further elucidated.
