# Corrigendum: Review of phytomedicine, phytochemistry, ethnopharmacology, toxicology, and pharmacological activities of Cymbopogon genus

**DOI:** 10.3389/fphar.2022.1109233

**Published:** 2022-12-07

**Authors:** Jonnea Japhet Tibenda, Qiong Yi, Xiaobo Wang, Qipeng Zhao

**Affiliations:** ^1^ School of Pharmacy, Key Laboratory of Hui Ethnic Medicine Modernization, Ministry of Education, Ningxia Medical University, Yinchuan, China; ^2^ Meishan Traditional Chinese Medicine Hospital, Meishan, China; ^3^ Research Institute of Integrated TCM and Western Medicine, Chengdu University of Traditional Chinese Medicine, Chengdu, China

**Keywords:** Cymbopogon, phytomedicine, phytochemistry, essential oils, pharmacological activity

In the published article, there was an error in **Affiliation 1**. Rather than “Key Laboratory of Hui Ethnic Medicine Modernization, School of Pharmacy, Ministry of Education, Ningxia Medical University, Yinchuan, China,” it should be “School of Pharmacy, Key Laboratory of Hui Ethnic Medicine Modernization, Ministry of Education, Ningxia Medical University, Yinchuan, China.”

The authors apologize for this error and state that this does not change the scientific conclusions of the article in any way. The original article has been updated.

